# Healthy Moves to Improve Lifestyle Behaviors of Cancer Survivors and Their Spouses: Feasibility and Preliminary Results of Intervention Efficacy

**DOI:** 10.3390/nu13124460

**Published:** 2021-12-14

**Authors:** Cindy L. Carmack, Nathan H. Parker, Wendy Demark-Wahnefried, Laura Shely, George Baum, Ying Yuan, Sharon H. Giordano, Miguel Rodriguez-Bigas, Curtis Pettaway, Karen Basen-Engquist

**Affiliations:** 1 Department of Palliative Care, Rehabilitation & Integrative Medicine, The University of Texas MD Anderson Cancer Center, Houston, TX 77030, USA; 2 Moffitt Cancer Center, Tampa, FL 33612, USA; Nathan.Parker@moffitt.org; 3 Department of Nutrition Sciences, The University of Alabama at Birmingham, Birmingham, AL 35294, USA; demark@uab.edu; 4 Mental Health Care Line, Michael E. DeBakey Veterans Affairs Medical Center, Houston, TX 77030, USA; Laura.Shely@va.gov; 5 Department of Behavioral Science, The University of Texas MD Anderson Cancer Center, Houston, TX 77030, USA; gpbaum@mdanderson.org (G.B.); kbasenen@mdanderson.org (K.B.-E.); 6 Department of Biostatistics, The University of Texas MD Anderson Cancer Center, Houston, TX 77030, USA; yyuan@mdanderson.org; 7 Department of Health Services Research, The University of Texas MD Anderson Cancer Center, Houston, TX 77030, USA; sgiordan@mdanderson.org; 8 Department of Colon & Rectal Surgery, The University of Texas MD Anderson Cancer Center, Houston, TX 77030, USA; mrodbig@mdanderson.org; 9 Department of Urology, The University of Texas MD Anderson Cancer Center, Houston, TX 77030, USA; cpettawa@mdanderson.org

**Keywords:** behavior change, diet, physical activity, couples, telehealth counseling

## Abstract

Spouses offer a primary source of support and may provide critical assistance for behavior change. A diet-exercise intervention previously found efficacious in improving cancer survivors’ lifestyle behaviors was adapted to utilize a couples-based approach. The aims were to test the feasibility of this couples-based (CB) intervention and compare its efficacy to the same program delivered to the survivor-only (SO). Twenty-two survivor-spouse couples completed baseline assessments and were randomized to the CB or SO interventions. The study surpassed feasibility benchmarks; 91% of survivors and 86% of spouses completed a 6-month follow-up. Survivors and spouses attended 94% and 91% of sessions, respectively. The SO survivors showed significant improvements on the 30-s chair stand and arm curl tests, weight, and fruit and vegetable (F and V) consumption. The CB survivors showed significant improvements on the 6-min walk and 2-min step tests, body weight, and fat and F and V consumption. Improvement in the 30-s chair stand and arm curl tests was significantly better for SO survivors. The SO spouses showed no significant changes in outcome measures, but the CB spouses showed significant improvements in moderate-to-strenuous physical activity, weight, and fat and F and V consumption. Weight loss was significantly greater in CB spouses compared to SO spouses. Findings demonstrate feasibility, warranting further investigation of CB approaches to promote lifestyle change among cancer survivors and spouses.

## 1. Introduction

Roughly half a century ago, the nation’s War on Cancer was launched and has resulted in major increases in survival through improvements in early detection and treatment [1]. There are now over 17 million cancer survivors in the US alone, comprising roughly 4% of the population [2]. While many survivors have been definitively treated for cancer, they remain at risk for recurrence and are at an increased risk for second cancers, cardiovascular disease, and diabetes [3]. Many survivors also experience lingering effects of cancer and its treatment, including fatigue, psychological distress, and accelerated functional decline [3,4]. Collectively, these health conditions impose considerable costs. The total economic burden of cancer was previously estimated at $263.8 billion with $20.9 billion and $140.1 billion from indirect morbidity costs (lost productivity due to illness) and indirect mortality costs (lost productivity due to premature death), respectively [5]. Given the burgeoning number of survivors and their potential impact on the health care system, improving their health status is a national priority [6]. The proposed study targets survivors of breast, prostate, and colorectal cancers because they are the largest segment of cancer survivors where survival rates currently exceed 90% [7].

Research in cancer survivors has shown that interventions promoting a healthy weight, a healthy diet, and increased physical activity improve quality of life (QOL), physical functioning and overall health status, and reduce the risk of chronic disease [8,9,10] and possibly recurrence [11], and improve survival [12,13]. It is recommended that cancer survivors achieve and maintain a healthy weight, accumulate at least 150 min of moderate physical activity per week, and consume a healthy plant-based diet [10,13,14]. However, a study of 3367 racially- and ethnically-diverse cancer survivors identified through the National Health Interview Survey indicates that roughly 70% of survivors are overweight or obese, and over 80% do not meet the guidelines for physical activity or fruit and vegetable (F and V) consumption [15]. While cancer survivors in general have similar and equally high prevalence rates for physical inactivity, poor diets, and obesity as those without cancer [3,16], their risk for developing co-morbidities and downstream costly events that result from interactions between their cancer, its treatment, and these lifestyle factors results in a significant burden. As such, interventions that target diet, physical activity, and weight management are essential.

Research is ongoing to explore how best to deliver lifestyle interventions for cancer survivors [17,18,19]. Successful behavior change interventions integrate theory to maximize effectiveness. Social Cognitive Theory (SCT) [20], one of the most robust theories of behavior change, posits that behavior is influenced by expectations formed through direct and observed experiences, which includes expectations about the confidence (self-efficacy) in performing these tasks successfully. Behavior also is influenced by goals, both proximal and distal, and by barriers to performance [21]. In the behavior change process, change is more likely when: (1) Behaviors are successfully performed independently; (2) Support is received from others who express confidence in that behavior change and provide feedback on performance; and (3) Desired behaviors are then modelled by others [20,22]. Thus, an integral part of SCT is the role that social relationships have on behavior change.

A recent scoping review by Ellis et al. [23] calls for interventions that capitalize on existing support networks that are conducted within the family context to promote healthy behaviors not only among cancer survivors, but also among their family members. A couples-based (CB) intervention is consistent with this call and also the tenants of SCT. This format encourages couples to model healthy eating and physical activity for each other; observing the other’s success can increase one’s confidence. In the process, couples learn to provide one another with support and feedback regarding goal setting and to work together to overcome barriers. Indeed, couples report that having their partner perform and model goal behaviors, join in health discussions, and provide emotional support encourages their own behavior change [24]. Unfortunately, enlisting the spouse only as a supporter may result in negative consequences, because even well-intentioned spouses may offer assistance in ways that appear controlling or over-protective, rather than supportive. As such, interventions addressing behavior change for both members of the couple and promoting shared goals, conjoint coping, and mutual support may be more effective than those targeting the individual cancer survivor [22]. This approach capitalizes on the strength of the spousal bond and embraces the recommendations that family members also follow the American Cancer Society guidelines for nutrition and physical activity [13]. Ultimately, both partners may benefit by reducing disease burden for survivors (tertiary prevention) [13] and the risk of cancer and other diseases in spouses (primary prevention) [25]. While recent pilot trials have included spouses in such interventions [26,27], none have focused on changing multiple health behaviors and none have compared their efficacy to a survivor only multiple health behavior change approach in which the health behavior outcomes of both the survivor and their spouse are examined.

The aims of the present study were to conduct a pilot trial to test the feasibility of a CB multi-behavior change program (diet and physical activity) and to compare its efficacy to the same program delivered to the survivor only (SO) in 22 survivors of breast, prostate, and colorectal cancer and their spousal partners, both of whom were identified as having poor health behaviors. The hypothesis was that survivors randomized to the CB format compared to the SO format would show favorable changes in physical activity, physical performance, body weight status, body composition, and diet. Likewise, and additionally, spouses would show greater changes in outcomes with the CB format compared to the SO format.

## 2. Materials and Methods

### 2.1. Study Overview

This study employs a 2-arm, single-blinded, randomized controlled trial (RCT) that evaluated a 6-month diet and exercise intervention delivered in either a CB or SO format. All participants completed assessments at baseline and 6 months (post-intervention).

### 2.2. Participant Eligibility

Eligibility for the survivor included: (1) Diagnosis of loco-regional breast cancer (Stages 0-IIIA), prostate cancer (Stages I-II), or colorectal cancer (Stages I-II); (2) Completion of primary cancer treatment and at least 3 months from surgery; (3) No history of other cancers (excluding non-melanoma skin cancer); (4) <150 min of moderate-to-vigorous intense physical activity (PA) per week; (5) Fruit and vegetable (F and V) intake <7 servings/day for women or <9 servings/day for men; (6) Age 18 years or older; (7) Able to read and speak English; (8) Living within the Houston area (Harris or a contiguous county); (9) No pre-existing medical conditions that precluded adherence to an unsupervised PA program or high fruit and vegetable (F and V) diet [28]; (10) Having a spouse or significant other with whom they have resided for at least 1 year (includes heterosexual and same-sex couples); (11) Able to provide informed consent; and (12) Has access to a computer with high-speed internet.

Eligibility for the spouse included criteria 4–12 listed above. Exclusion criteria for survivor or spouse included using a walker or wheelchair/scooter, being pregnant, or reporting any conditions that are listed on the Physical Activity Readiness Questionnaire (PAR-Q) [29].

### 2.3. Recruitment and Screening Procedures

This research was approved by The University of Texas MD Anderson Institutional Review Board. Participants who participated in another MD Anderson approved protocol and who indicated they would like to be contacted for future lifestyle trials were recruited for this study. First, potential participants were contacted and verified for eligibility following verbal consent for screening, including cancer diagnosis and treatment status, and having lived with a spouse or significant other for at least 1 year. Next, they were screened for current physical activity using the Godin Leisure Time Exercise Questionnaire (GLTEQ) and F and V intake using the 2009 Texas Behavioral Risk Factor Surveillance System (BRFSS) dietary questions, as well as whether there were any pre-existing medical conditions that precluded their participation using the PAR-Q. Survivors endorsing any item on the PAR-Q were required to have a medical release from their physician to clear them for participation in the study. Following permission to contact their spouses, an identical process was then used to solicit spousal interest, gain verbal consent, and screen for eligibility. For this study, both survivors and their spouses had to be eligible and provide written consent for participation. For all survivors and/or spouses not interested in participating, information regarding reasons for refusal was collected.

### 2.4. Study Group Assignment

After completing baseline assessments, participants were assigned to 1 of the 2 study conditions using a form of adaptive allocation referred to as minimization [30]. The following survivor variables were used to ensure balance across study group assignment: baseline physical activity, baseline diet quality, age, race, gender, and marital quality. Spousal factors were not included because doing so would likely be redundant given the literature showing a strong concordance between spousal health behavior [31]. Group assignment was conducted separately by disease site. Minimization has been used successfully in previous trials resulting in a good group balance [32,33].

### 2.5. Study Conditions

The diet and exercise intervention, based on Social Cognitive Theory, was a tailored correspondence and web-based counseling regimen initially developed and proven efficacious for breast, prostate, and colorectal cancer survivors by Demark Wahnefried and colleagues in Reach out to EnhaNcE Wellness (RENEW) [34]. The behavioral goals were for participants to engage in 15 min of strength exercise every other day, ≥30 min of walking or other moderate-intensity exercise on 5 or more days per week, and consume a diet of ≥7 F and V servings/day for women or ≥9 F and V servings/day for men and ≤7% of total calories from saturated fat [35]. It also encouraged weight management; for those with a BMI ≥ 25 kg/m^2^, a healthy weight loss goal of 1–2 lbs. per week (a loss of 5% body weight was used as a goal over the course of the 6-month study period) was encouraged. To adapt to the 6-month timeline, the RENEW intervention materials were modified such that they could be delivered within the study period and included materials directed toward the spouse. For survivors randomized to the survivor-only arm, the materials were similar; however, there was no reference to working with a spouse on behavior change efforts. Survivors (and their spouses if randomized to the CB arm) were provided with a tailored workbook and 3 tailored print newsletters over the 6-month study period. All print materials provided motivational messages tailored on stage of readiness [36] that accompanied illustrations of current behaviors in relation to national guidelines; progress reports depicted headway toward goals (which were incrementally set) and reinforced. The SO survivors and CB survivors and their spouses also received 9 web-based video counseling sessions; in the unforeseen event that there were problems connecting to the session online, participants had the option of receiving these sessions by telephone. The first 3 sessions were weekly; sessions changed to every other week after session 3, and then monthly after session 5. The counselor had a master’s degree in Marriage and Family Counseling and was supervised by a licensed clinical psychologist with expertise in counseling and health behavior change. Each counseling session focused on specific cognitive-behavioral strategies for healthy behavior change. All sessions also emphasized SCT concepts, such as self-monitoring and incremental goal setting and specific session topics included the following: problem-solving; relapse prevention; goal-setting; cognitive restructuring; and time management [20]. Skills practice was assigned as homework to be reviewed in subsequent sessions. For both study conditions, assessments of adverse events were conducted at the start of each counseling session. Finally, participants in each arm were provided with the following materials: Therabands^®^; T-Factor 2006© Guide to the Fat Content of Foods; portion plate; pedometer; web camera; headset; and log books to track their exercise and diet behaviors. In addition to the intrapersonal (individual) cognitive-behavioral strategies oriented toward one’s own behavior change, participants in the CB intervention learned interpersonal cognitive-behavioral skills, including communal coping, joint problem-solving, and healthy communication. They also received the counseling sessions together as a couple.

### 2.6. Assessment Procedures

All participants (survivors and spouses) were assessed in-person at MD Anderson at baseline and at a 6-month follow-up. Assessment personnel were blinded to the participant’s study condition. Accrual, attrition, and patient satisfaction served as the primary endpoints of this feasibility study, and other outcomes, such as physical activity, physical performance, weight status, body composition, and dietary intake were also assessed. Each survivor and spouse who completed the baseline and 6-month assessments received compensation in the form of $25 gift cards: one following each completed assessment (up to $100 per couple). Participants also received relevant assessment results at the end of the study. If one member of the survivor-spouse dyad dropped out, the remaining member of the couple could continue.

### 2.7. Measures

To address the study aims, feasibility, and exploratory outcome measures were collected:

Feasibility measures included accrual, attrition, participant views on intervention acceptability, and the monitoring of adverse events. For recruitment, the number of participants contacted about the study, who were eligible and who consented to participate, were tracked. Retention was calculated as the percentage of participants assessed at baseline who completed the 6-month assessments. Drop outs were tracked by study condition. Session attendance was monitored to measure exposure. Intervention acceptability was assessed by asking participants, “Would you recommend this program to other cancer survivors?” at the 6-month follow-up. Possible responses included “Yes,” “Maybe,” and “No.” Responses were compiled across study conditions.

Exploratory outcome measures included physical activity, physical performance, body composition, weight, and diet.

Physical activity was assessed with the 3-item modified version of the Godin Leisure Time Exercise Questionnaire to ascertain self-reported moderate and strenuous leisure time exercise [37]. To provide an objective measure of physical activity, for 1 week before the baseline and the 6-month assessments, the participants also wore a programmed Actigraph accelerometer (Fort Walton Beach, FL, USA). Accelerometers were downloaded according to the manufacturer’s instructions and as per the previous studies of Basen-Engquist and colleagues [38].

Physical performance was measured using a variety of tests that assessed endurance, strength, and agility. The 6-min walk test and 2-min step test were used as measures of aerobic function. The 6-minute walk test has been validated in older adults by comparing it to a treadmill walking test measuring the time to get to 85% of an age-adjusted maximum heart rate [39]. The 2-min step test is self-paced and assesses the number of times within 2 min a participant can step in place raising the knees to a height halfway between the iliac crest and mid-patella. This test correlates moderately with common measures of aerobic capacity and is low risk [40]. For lower body strength, a 30-s chair-stand test was used [41]. For upper body strength and functionality, the timed arm curl task was used, taking into account that this test has been shown to be better tolerated than maximum-grip strength for participants with arthritis [41]. To assess agility and dynamic balance, an 8-foot up-and-go assessment was used. The task is a composite measure involving dynamic balance, power, and agility. The test is a modification of the 3-m time up-and-go test [42]. The modification to 8 feet is to increase the feasibility of administering the tests in areas with limited space, including home settings [41]. The height at baseline (for BMI calculation) and body weight were measured using a stadiometer and electronic scale, respectively.

Diet was assessed with the Automated Self-administered 24-h Dietary Recall (ASA24) to document the participant’s food intake for a total of 24 h. Two interviews were obtained, 1 for a weekday and 1 for a weekend day [43]. The foods selected by the participants are from the USDA’s Food and Nutrient Database for Dietary Studies’ (FNDDS) most up-to-date database. Participants in both groups completed this assessment 1 week before their baseline and 6-month assessments. F and V intake and % of calories from dietary fat were extracted from the ASA24 output and averages for the 2-day recalls were taken at each of the time points.

Spouse exploratory outcome measures included the same measures of physical activity, physical performance, diet, and weight. These were assessed at the same time points as survivors.

Finally, demographic/medical questions for survivors were collected at baseline and included age, sex, race/ethnicity, education level, employment status, cancer type/stage, and treatment types. Demographic data for spouses included age, sex, and race/ethnicity.

### 2.8. Data Analysis

Summary statistics were calculated for demographic and clinical characteristics for the study population by study condition.

Feasibility was determined by 3 criteria: (1) The completion of accrual within 1 year; (2) An attrition rate of 20% or less; and (3) No occurrence of serious adverse events that are directly attributable to the intervention.

For the exploratory outcome measures, we calculated the means and standard deviations. Prior to computing the sum for moderate-to-strenuous physical activity, moderate and strenuous physical activity variables were each truncated at 420 min per week. Paired t-tests or Wilcoxon signed-rank tests were used to assess within group differences between the 6-month and baseline measurements. We also calculated the difference between the 6-month measurement and baseline and compared it between groups using a 2-sample t-test or Mann-Whitney U test. To estimate the effect of the study group assignment (CB arm relative to SO arm) on changes in exploratory outcome variables, multivariable linear regression models were fit for each outcome. The covariates included in multivariable linear regression models were selected based on the univariates analysis with *p* < 0.05.

## 3. Results

### 3.1. Participant Characteristics

Table 1 displays clinicodemographic characteristics of survivors and spouses randomized to the SO and CB groups. On average, survivors were in their early-to-mid 60s, and slightly more were female. Survivors were predominantly white, and the vast majority had at least some college-level education. Nearly one-third of survivors were employed full time, and nearly one-third classified themselves as a homemaker/volunteer, while slightly more than one-third were retired. The average BMI among survivors was in the overweight range. All female participants were breast cancer survivors, whereas most male participants were prostate cancer survivors. In terms of cancer treatment, most survivors had undergone surgery, radiation therapy, and hormonal therapy, but fewer than half of the survivors had undergone chemotherapy. There were no significant differences in clinicodemographic characteristics between survivors randomized to the SO and CB conditions (all *p*-values > 0.05). As published previously [44], survivors who enrolled in the study were younger and consumed less energy from fat than survivors who were screened but did not enroll.

The average age of spouses was similar to that of survivors. The majority of spouses were male and white. The average BMI of spouses also was in the overweight range, though higher than survivors. There were no significant differences in demographic characteristics between spouses randomized to the SO and CB conditions (all *p*-values > 0.05).

### 3.2. Feasibility Measures

**Figure 1:**
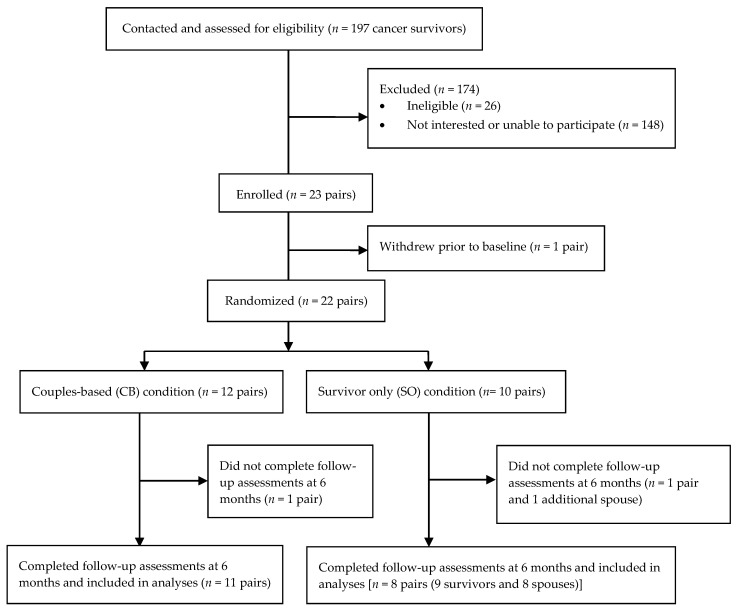
CONSORT flow diagram from recruitment to study completion.

Recruitment spanned 15 months, with 22 survivors and 22 spouses enrolling between July 2011 and September 2012. One hundred ninety-seven survivors were contacted, and 22 survivors (11.2%) enrolled and completed baseline assessments (Figure 1). One couple enrolled but did not complete baseline assessments. The overall enrollment rate was 12.7%.

Nine-of-ten survivors in the SO group (90.0%) completed the 6-month follow-up measures, and 11-of-12 survivors in the CB group (92.7%) completed the 6-month follow-up measures. Thus, the attrition rate among survivors was 9.1%. Eight-of-ten spouses in the SO condition (80%) and 11-of-12 spouses in the CB condition (92.7%) completed the 6-month follow-up measures, resulting in a 13.6% attrition rate among spouses. The overall attrition rate, regardless of survivor status or study condition, was 11.4%.

Survivors in the SO condition attended an average of 97% of sessions, and survivors in the CB condition attended an average of 92% of sessions. Combining both groups, survivors attended 94% of sessions with no significant differences between study conditions. Spouses (CB condition only) attended an average of 91% of sessions.

In terms of intervention acceptability, 12 survivors (6 CB and 6 SO) and 5 spouses (4 CB and 1 SO) completed the follow-up question asking whether they would recommend the program to other cancer survivors. Among CB survivors, 5 (83%) responded, “Yes,” and 1 (17%) responded, “No.” Among SO survivors, 4 (67%) responded, “Yes,” and 2 (33%) responded “Maybe.” Among spouses, all 4 spouses from the CB group (100%) responded, “Yes” and the 1 spouse from the SO group responded “Maybe.”

There were no intervention-related adverse events.

### 3.3. Exploratory Outcome Measures

Table 2 display secondary outcomes including self-reported and accelerometer-based physical activity, physical functioning, weight status, body composition, and diet at study time points for survivors and spouses. The *p*-values indicating the significance of change within the study arm and the significance of the difference in change between the study arms is also presented.

There were no significant changes in either self-reported or accelerometer measures or physical activity between study time points among survivors randomized to the SO vs. CB conditions, and there were no significant differences in physical activity change between these groups.

Despite no differences in physical activity, there were significant changes in physical performance from baseline to the 6-month follow-up. Survivors randomized to the CB condition showed significant improvement in both the 6-min walk test and the 2-min step test at 6 months, whereas survivors randomized to the SO condition showed no significant change in these measures. No significant between-arm differences were detected for either of these measures. Survivors randomized to the SO condition showed significant improvement in the 30-s sit-to-stand test and in the arm curl test, whereas survivors in the CB condition showed no significant change in either of these tests. Improvements in these tests were significantly better for the SO vs. the CB arms.

Survivors in both the SO and CB arms demonstrated significant weight loss over the 6-month period with no between-arm differences in weight loss noted.

The SO arm survivors reported significantly higher F&V consumption at 6 months compared to baseline, as did CB arm survivors. Survivors in the CB arm also had significant decreases in saturated fat consumption. There were no significant between-arm differences in change scores for any of the dietary variables.

Spouses randomized to the CB condition reported significantly higher strenuous + moderate physical activity at 6 months compared to baseline, but there was no significant change in this variable for spouses in the SO arm. There were no significant changes in either arm in accelerometer-measured physical activity. There were no significant differences between arms in the amount of change in either self-reported or accelerometer-measured physical activity.

There were no significant changes in physical performance measures from baseline to 6 months among spouses randomized to either study condition, and there were no significant differences in physical performance changes between groups. Spouses randomized to CB condition demonstrated significant weight loss at 6 months relative to baseline. Spouses randomized to the SO condition showed no significant change in weight, and there was no significant difference in weight change between groups.

Spouses randomized to the CB condition showed significantly reduced consumption of total fat and saturated fat and significantly increased consumption of F&Vs at 6 months relative to baseline. In contrast, spouses randomized to the SO condition showed no significant changes in total fat, saturated fat, or F&V consumption from baseline to 6 months. There were no significant differences in 6-month changes in dietary variables between spouses randomized to SO vs. CB study conditions.

Table 3 displays multivariable linear regression models estimating the effect of study groups on exploratory outcome measures for cancer survivors and spouses. Based on bivariate correlations with outcome variable change scores, the following variables were included as covariates in the models: baseline value of the outcome of interest, ethnicity (white vs. non-white), and BMI. With randomization to the SO condition as the comparison group, randomization to the CB condition showed significant, negative associations with change in 30-s chair stand repetitions (B = −2.7, *p* = 0.04) and arm curls (B = −4.5, *p* = 0.02) among survivors. Among spouses, randomization to the CB condition showed a significant, negative association with change in vigorous physical activity (B = −4.08, *p* = 0.02).

## 4. Discussion

Few studies have examined the feasibility or outcomes of spouse-based interventions to improve lifestyle behaviors among cancer survivors [41]. This study examined the feasibility of a CB intervention to improve diet and physical activity among cancer survivors and their spouses, and also compared differences in exploratory outcomes between survivors and spouses randomized to the CB intervention and those randomized to an SO intervention.

As hypothesized, the intervention was indeed feasible, with survivors and spouses surpassing metrics for retention and intervention session attendance. The enrollment rate for this study was 12.7%, which is typical of interventions targeting diet and physical activity among cancer survivor-partner dyads [27,45], particularly when survivors are not referred directly by oncologists involved in the survivors’ care. Furthermore, retention and adherence in the current trial were strong. The combined attrition rate of 11.4% among all participants in this study is similar to or exceeds those reported from other studies involving cancer survivor-caregiver or partner dyads [27,45,46]. Moreover, the high attendance, which ranged from 91–97%, exceed those reported in recent studies involving exercise for cancer survivor-partner dyads [27,46], further highlighting the feasibility of this intervention. Participants experienced no intervention-related adverse events thus safety, an important outcome to establish feasibility, was obviated as a concern. Both survivors and spouses tended to rate the program favorably, with 92% of survivors and 80% of spouses responding that they would recommend participating to other cancer survivors.

Cancer survivors and their partners tend to struggle to consume healthy diets and engage in sufficient exercise, [15] so establishing intervention feasibility, as we did in this study, is a critical first step. The high rates of retention and adherence observed in this study highlight the importance and benefits of couples embarking on paths to improve eating and physical activity habits together. Similarly, low rates of attrition and high rates of adherence between survivors randomized to both study arms suggest that the two groups may have inspired similar levels of motivation to participate and complete the intervention. Attending intervention sessions and following-up to measure progress set the stage for developing positive health behaviors as a couple, with survivors and spouses each taking active roles in supporting one another’s efforts to improve health. The observed enrollment rate, though typical in the realm of behavior change interventions for cancer survivors, leaves significant room for improving intervention reach. Studies that rely on treating oncologists to refer cancer survivors to behavior change interventions tend to demonstrate higher enrollment rates [27]; this highlights the importance of integrating lifestyle improvement programming into standard care for cancer care and survivorship. Future trials involving dyadic interventions to improve diet and physical activity among cancer survivors and their spouses may benefit from involving oncology providers directly in referral pathways.

In addition to intervention feasibility, our study provides some evidence of intervention benefits for both cancer survivors and their partners. Survivors in both the CB and SO groups improved health behaviors and related outcomes with between-group comparisons demonstrating few differences. However, spouses in the CB intervention demonstrated significant improvements in health behaviors and related outcomes, while those examined as part of the SO group (i.e., did not receive an intervention) demonstrated none. Though samples were small, these findings suggest that CB interventions may help enhance delivery to some cancer survivors and may provide an important opportunity for behavior change among spouses.

Recently published studies involving lifestyle interventions for dyads featuring cancer survivors and their caregivers or family members have demonstrated mixed results. Kamen et al. found no significant improvements in physical activity (steps per day) among cancer survivors or caregivers (95% of whom were spouses/partners) following a 6-week exercise intervention, and there was no significant difference between participants who were randomized to engage with their caregivers and those randomized to individual intervention [47]. In contrast, Demark-Wahnefried et al. found significant improvements in physical activity, fitness, and anthropometrics among breast cancer survivors and their adult daughters enrolled in team and individual lifestyle interventions, but no significant differences between intervention formats [45]. To date, studies of dyadic lifestyle interventions for cancer survivors and their caregivers or partners, including the current study, have focused on understanding intervention feasibility. As such, they likely lack statistical power to detect true differences in behaviors or outcomes between groups of survivors and partners receiving the intervention as pairs and those receiving interventions individually.

This study has important strengths and limitations. Strengths include a strong, randomized study designed to compare intervention feasibility and primary and secondary outcomes between study groups. Primary outcomes included valid measures of diet, and both objective and self-reported physical activity, and secondary outcomes included valid and objective measures of physical performance, body composition, and anthropometrics. Intervention adherence and retention were very strong and similar between study groups, helping to limit concerns about intervention fidelity, attrition bias, or missing data. The enrollment rate of 12.7%, though on par with dyadic lifestyle interventions for cancer survivors, suggests that those who actually participated may have been particularly motivated to engage in a lifestyle intervention with their spouses. Future efforts to enroll cancer survivors in dyadic lifestyle interventions may benefit from directly involving clinicians in recruitment efforts. Though the randomized design helps ensure that there were no systematic differences in motivation between groups, the overall findings of the study may not generalize to the broader population of cancer survivors, many of whom may be less motivated to make healthy lifestyle changes. Generalizability may also be limited by the relatively homogeneous, sociodemographic profile of study participants, as most participants were non-Hispanic white and well-educated, and had an opposite sex partner/spouse. Future research should recruit more diverse samples; in particular the needs of couples who are not heterosexual or are from different racial/ethnic groups needs focused study. The study had a number of secondary outcomes, multiple testing, and potentially spurious significant differences, which is another important limitation. Finally, as the primary study purpose was to examine the feasibility of the lifestyle intervention, the lack of statistical power to detect differences between groups in outcome measures was a primary limitation. The promising feasibility metrics in adherence and retention we observed in this study, coupled with plans to enhance recruitment strategies to enroll a larger and potentially more generalizable population, lend promise to a large, impactful RCT examining differences in outcomes by intervention delivery strategies.

## 5. Conclusions

Few studies have incorporated spouses in behavioral interventions for cancer survivors, despite the importance of relationships between cancer survivors and their loved ones in survivorship and the potential to broaden the impact of positive behavior change [48]. Cancer diagnosis, treatment, and survivorship expose both parties of caregiving relationships to stressful events that can impact health and well-being. Given the many opportunities for important family decisions throughout cancer survivorship, these circumstances may be particularly opportune times for lifestyle interventions. The well-being of cancer survivors and their caregivers tend to covary over time throughout cancer treatment and survivorship, and the positive role modeling and social support for behavior change that may result from dyadic interventions can provide mutual benefits for both cancer survivors and their spouses in this context [27,49,50]. Spousal support plays an important role in diet, as couples generally rely on the same strategies for food procurement and preparation [51,52]. Improvements detected among both cancer survivors and spouses in the CB group may reflect this important dynamic of dietary habits for couples and highlights the window of opportunity to impact both members’ diet with dyadic lifestyle interventions for cancer survivors. Study findings showing improvements in physical activity, physical performance, anthropometrics, and diet among cancer survivors in both groups and spouses in the CB group are promising, albeit preliminary. It will be important for a future trial to be powered to compare outcomes between couples randomized to receive the intervention together and separately. Overall, the findings from this study suggest that dyadic lifestyle multiple behavior change interventions are promising for both cancer survivors and their spouses, and they may provide a valuable strategy to broaden and deepen the impact of improving health during cancer survivorship.

## Figures and Tables

**Table 1:** Clinic demographic characteristics of cancer survivor and spouse participants by study condition.

Characteristic	Cancer Survivors				Spouses			
	Overall (*n* = 22)	Survivor-Only Condition (*n* = 10)	Couples-Based Condition (*n* = 12)	*p* ^a^	Overall (*n* = 22)	Survivor-Only Condition (*n* = 10)	Couples-Based Condition (*n* = 12)	*p* ^a^
Age (years), mean (SD)	64.1 (10.8)	62.4 (11.4)	65.5 (10.5)	0.5	63.4 (8.2)	63.1 (9.0)	63.4 (7.8)	0.9
Sex				0.7				>0.9
Male, *n* (%)	10 (45.5)	4 (40.0)	6 (50.0)		13 (59.1)	6 (60.0)	7 (58.3)	
Female, *n* (%)	12 (54.5)	6 (60.0)	6 (50.0)		9 (40.9)	4 (40.0)	5 (41.7)	
Race/Ethnicity				0.1				0.6
Hispanic, *n* (%)	4 (18.2)	3 (30.0)	1 (8.3)		3 (13.6)	1 (10.0)	2 (16.7)	
Non-Hispanic Black, *n* (%)	1 (4.5)	1 (10.0)	0 (0.0)		2 (9.1)	1 (10.0)	1 (8.3)	
Non-Hispanic White, *n* (%)	16 (72.7)	5 (50.0)	11 (91.7)		16 (72.7)	7 (70.0)	9 (75.0)	
Other, *n* (%)	1 (4.5)	1 (10.0)	0 (0.0)		1 (4.5)	1 (10.0)	0 (0.0)	
Education, *n* (%)				0.7				-
High school diploma/GED	2 (9.1)	0 (0.0)	2 (16.7)		-	-	-	
Some college or 2-year degree	6 (27.3)	3 (30.0)	3 (25.0)		-	-	-	
Bachelor’s degree	8 (36.4)	3 (30.0)	5 (41.7)		-	-	-	
Advanced degree	6 (27.2)	4 (40.0)	2 (16.6)		-	-	-	
Employment Status, *n* (%)				0.2				-
Full Time	6 (27.3)	1 (10.0)	5 (41.7)		-	-	-	
Part Time	2 (9.1)	1 (10.0)	1 (8.3)		-	-	-	
Retired	8 (36.4)	6 (60.0)	2 (16.7)		-	-	-	
Homemaker or volunteer	6 (27.3)	2 (20.0)	4 (33.3)		-	-	-	
Weight (kg), mean (SD)	76.4 (19.4)	70.4 (13.8)	81.4 (22.4)	0.2	85.7 (22.6)	80.8 (12.8)	89.8 (28.3)	0.4
BMI (kg/m^2^), mean (SD)	27.7 (6.4)	25.4 (3.8)	29.7 (7.6)	0.1	29.6 (5.8)	28.2 (3.9)	30.7 (7.0)	0.3
Cancer type, *n* (%)				0.6				-
Breast	13 (59.1)	6 (60.0)	7 (58.3)		-	-	-	
Prostate	8 (36.3)	4 (40.0)	4 (33.3)		-	-	-	
Colorectal	1 (4.5)	0 (0.0)	1 (8.3)		-	-	-	
Surgery, *n* (%)				0.2				-
No	2 (10.5)	0 (0.0)	2 (22.2)		-	-	-	
Yes	17 (89.5)	10 (100.0)	7 (77.8)		-	-	-	
Chemotherapy				>0.9				-
No	11 (57.9)	6 (60.0)	5 (55.6)		-	-	-	
Yes	8 (42.1)	4 (40.0)	4 (44.4)		-	-	-	
Radiation therapy				>0.9				-
No	9 (45.0)	4 (40.0)	5 (50.0)		-	-	-	
Yes	11 (55.0)	6 (60.0)	5 (50.0)		-	-	-	
Hormonal therapy				>0.9				-
No	7 (38.9)	3 (33.3)	4 (44.4)		-	-	-	
Yes	11 (61.1)	6 (66.7)	5 (55.6)		-	-	-	
Other treatment				0.6				-
No	9 (75.0)	3 (60.0)	6 (85.7)		-	-	-	
Yes	3 (25.0)	2 (40.0)	1 (14.3)		-	-	-	

^a^ For difference between individual and couple condition.

**Table 2:** (**a**) Summary statistics for outcome measures among cancer survivors by study condition; (**b**) Summary statistics for outcome measures among spouses by study condition.

**(a)**										
		**Cancer Survivors**								
		**Survivor-Only Condition**				**Couples-Based Condition**				
**Outcome**	**Assessment**	***n***	**Mean**	**SD**	***p* ^a^**	***n***	**Mean**	**SD**	***p* ^a^**	***p* ^b^**
Self-reported moderate-to-strenuous PA (min/wk)	Baseline	10	176.0	138.7	0.3	12	96.0	116.6	0.8	0.6
	6 months	10	196.5	158.0		11	107.7	112.1		
MPA (min/wk)	Baseline	9	71.3	46.0	0.4	11	38.3	18.0	0.2	0.3
	6 months	8	90.0	28.8		7	57.0	25.3		
VPA (min/wk)	Baseline	9	6.0	7.7	0.9	11	0.3	0.7	0.1	0.7
	6 months	8	3.7	4.1		7	1.2	1.9		
MVPA (min/wk)	Baseline	9	77.3	48.7	0.5	11	38.6	18.2	0.2	0.7
	6 months	8	93.7	31.3		7	58.2	25.8		
6-min walk distance (m)	Baseline	10	514.7	68.4	0.2	12	443.0	87.2	<0.001	0.5
	6 months	9	580.8	100.1		11	531.2	81.9		
2-min step test (repetitions)	Baseline	10	79.8	27.9	0.3	12	82.0	16.3	0.01	0.5
	6 months	9	92.4	26.0		11	98.9	14.0		
30-Second Sit-to-Stand (repetitions)	Baseline	10	11.4	3.2	0.005	12	12.1	3.1	0.5	0.005
	6 months	9	15.0	5.1		11	12.8	3.1		
Arm Curls (repetitions)	Baseline	10	13.2	3.4	0.01	12	14.8	3.1	0.8	0.02
	6 months	9	17.6	5.8		11	15.6	4.4		
8 foot up-and-go time (s)	Baseline	10	6.3	1.9	0.3	12	7.0	1.5	0.7	0.5
	6 months	9	5.6	0.9		11	6.7	1.7		
Body weight (kg)	Baseline	10	70.4	13.8	0.02	12	81.4	22.4	0.01	0.5
	6 months	9	67.0	13.8		11	73.3	14.1		
Total fat consumption (g/day)	Baseline	10	72.9	19.0	0.5	12	76.7	24.6	0.07	0.5
	6 months	9	64.9	31.8		11	60.3	29.7		
Saturated fat consumption (g/day)	Baseline	10	21.1	7.1	0.6	12	24.5	10.5	0.03	0.2
	6 months	9	19.0	9.5		11	18.2	7.1		
Fruit and vegetable consumption (cups/day)	Baseline	10	2.5	1.3	0.02	12	2.6	1.3	<0.001	0.8
	6 months	9	4.4	2.0		11	4.5	1.6		
**(b)**										
		**Spouses**								
		**Survivor-Only Condition**				**Couples-Based Condition**				
**Outcome**	**Assessment**	***n***	**Mean**	**SD**	***p*** **^a^**	***n***	**Mean**	**SD**	***p*** **^a^**	***p*** **^b^**
Self-reported moderate-to-strenuous PA (min/wk)	Baseline	9	129.4	158.6	0.9	12	71.7	78.1	0.02	0.8
	6 months	9	120.0	157.9		10	124.5	47.9		
MPA (min/wk)	Baseline	9	80.3	47.8	0.6	12	52.0	36.1	0.2	0.06
	6 months	9	87.0	31.8		6	55.7	29.6		
VPA (min/wk)	Baseline	9	3.1	5.7	0.2	12	3.6	7.1	0.5	0.2
	6 months	9	6.1	12.3		6	5.5	13.0		
MVPA (min/wk)	Baseline	9	83.4	49.1	0.9	12	55.6	41.5	0.2	0.2
	6 months	9	92.9	30.8		6	61.2	40.1		
6-min walk distance (m)	Baseline	10	504.5	112.4	>0.99	12	491.3	131.2	0.5	0.6
	6 months	8	533.8	96.9		11	508.0	86.3		
2-min step test (repetitions)	Baseline	10	86.9	22.9	0.8	12	80.3	32.7	0.6	0.6
	6 months	8	86.0	18.5		11	84.0	17.5		
30-Second Sit-to-Stand (repetitions)	Baseline	10	11.9	4.8	0.6	12	12.0	3.8	>0.99	0.5
	6 months	8	12.1	6.6		11	12.4	3.9		
Arm Curls (repetitions)	Baseline	10	16.3	4.8	0.8	12	16.9	5.4	0.2	0.3
	6 months	8	16.8	4.8		11	15.7	4.8		
8 foot up-and-go time (s)	Baseline	10	7.1	2.6	0.3	12	7.6	4.9	0.3	0.2
	6 months	8	6.7	1.6		11	6.3	1.6		
Body weight (kg)	Baseline	10	80.8	12.8	0.7	12	89.8	28.3	0.03	0.05
	6 months	8	83.6	11.3		11	80.2	20.1		
Total fat consumption (g/day)	Baseline	9	58.9	26.3	0.2	12	85.9	38.8	<0.001	0.8
	6 months	8	51.1	27.3		11	63.2	26.6		
Saturated fat consumption (g/day)	Baseline	9	20.1	10.1	0.4	12	28.5	13.0	0.002	0.4
	6 months	8	18.0	10.9		11	19.0	8.7		
Fruit and vegetable consumption (cups/day)	Baseline	9	2.8	1.5	0.6	12	2.4	1.3	0.01	0.2
	6 months	8	3.3	1.3		11	3.3	1.4		

Abbreviations: PA = physical activity, MPA = accelerometer-measured moderate physical activity, VPA = accelerometer-measured vigorous physical activity, MVPA = accelerometer-measured moderate-to-vigorous physical activity, ^a^ for within-group differences between baseline and 6 months; ^b^ for differences in change from baseline to 6-months between study conditions.

**Table 3:** Multiple linear regression models estimating treatment effects.

		Cancer Survivors				Spouses			
	Effect	βeta	95% LB	95% UB	*p*-Value	βeta	95% LB	95% UB	*p*-Value
Self-reported moderate-to-strenuous PA (min/wk)	Condition (SO vs. CB)	−12.08	−161.14	136.99	0.866	46.76	−51.05	144.56	0.323
MPA (min/wk)	Condition (SO vs. CB)	−33.86	−68.27	0.56	0.053	−15.47	−40.55	9.61	0.196
VPA (min/wk)	Condition (SO vs. CB)	−2.26	−7.16	2.65	0.320	−4.08	−7.32	−0.83	0.019
MVPA (min/wk)	Condition (SO vs. CB)	−36.30	−72.95	0.34	0.052	−15.76	−43.91	12.38	0.237
6-min walk distance (meters)	Condition (SO vs. CB)	23.57	−58.61	105.75	0.550	1.15	−46.03	48.34	0.959
2-min step test (repetitions)	Condition (SO vs. CB)	11.98	−6.67	30.62	0.191	1.93	−10.75	14.61	0.749
30-Second Sit-to- Stand (repetitions)	Condition (SO vs. CB)	−2.71	−5.30	−0.12	0.042	1.43	−2.28	5.15	0.421
Arm curls (repetitions)	Condition (SO vs. CB)	−4.46	−8.18	−0.73	0.022	−1.47	−4.32	1.37	0.285
8 foot up-and-go time (seconds)	Condition (SO vs. CB)	0.65	−0.62	1.92	0.294	−0.67	−1.84	0.50	0.237
Body weight (kg)	Condition (SO vs. CB)	−0.63	−3.58	2.33	0.658	−3.66	−7.90	0.57	0.085
Total fat consumption (g/day)	Condition (SO vs. CB)	−5.08	−35.10	24.94	0.723	4.10	−14.74	22.93	0.646
Saturated fat consumption (g/day)	Condition (SO vs. CB)	−2.45	−10.37	5.46	0.519	−1.62	−9.40	6.17	0.661
Fruit and vegetable consumption (cups/day)	Condition (SO vs. CB)	0.30	−1.37	1.98	0.704	0.69	−0.30	1.67	0.156

Abbreviations: PA = physical activity, MPA = accelerometer-measured moderate physical activity, VPA = accelerometer-measured vigorous physical activity, MVPA = accelerometer-measured moderate-to-vigorous physical activity.

## Data Availability

Data supporting reported results can be accessed via the corresponding author.
